# Supplementary material for: Association between oral microbiome and seven types of cancers in East Asian population: a two-sample Mendelian randomization analysis
Source: Front Mol Biosci. 2023 Nov 21;10:1327893. doi: 10.3389/fmolb.2023.1327893 (PMC10702768; doi:10.3389/fmolb.2023.1327893)
Supplement: Supplementary file 1 [file DataSheet1.ZIP › Supplementary Materials/MR plots for tongue/tongue═╝/Ovarian cancer/pheno.2262_to_ovarian cancer_leave_one_out.pdf]

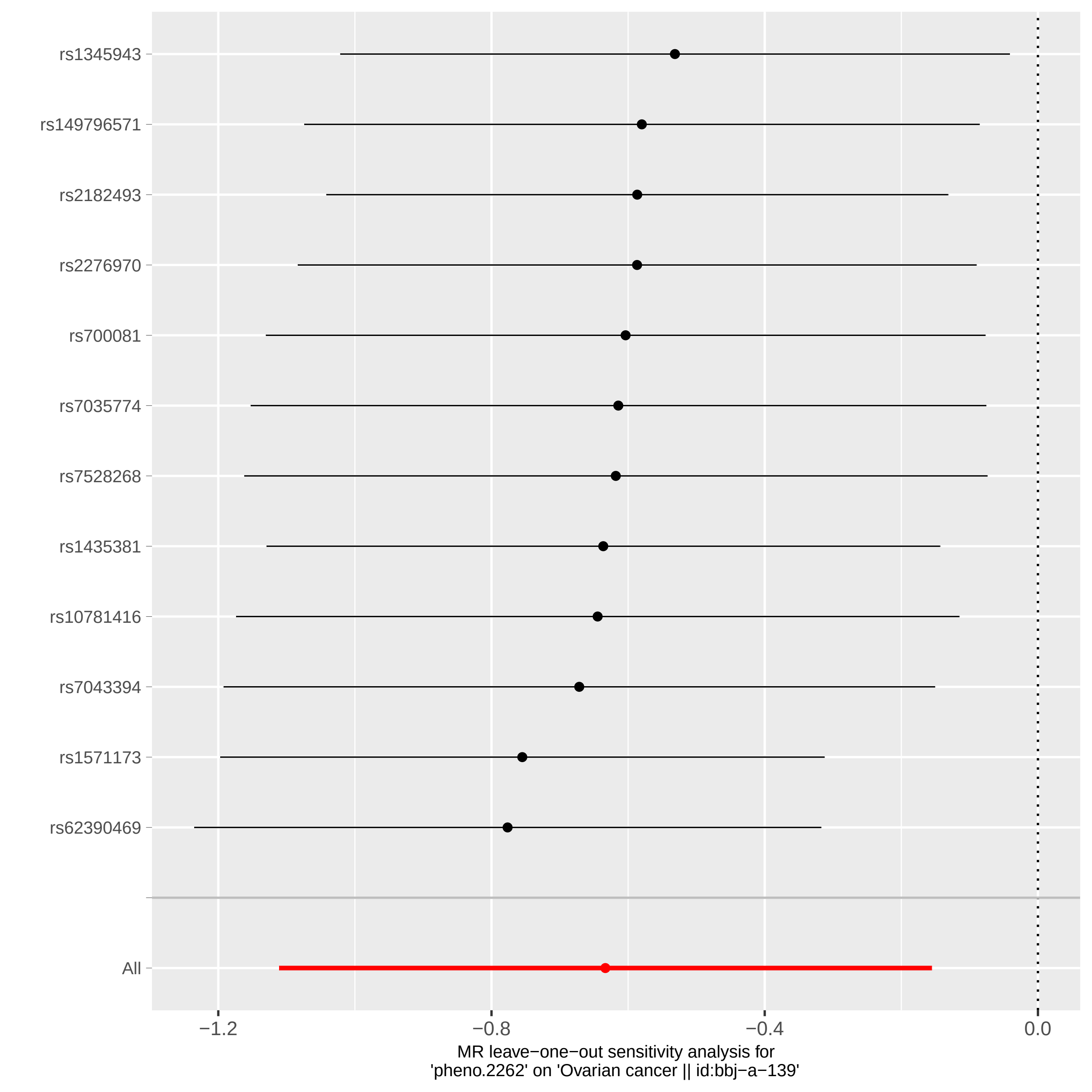

rs1345943
rs149796571
rs2182493
rs2276970
rs700081
rs7035774
rs7528268
rs1435381
rs10781416
rs7043394
rs1571173
rs62390469
All
−1.2
−0.8
−0.4
0.0
MR leave−one−out sensitivity analysis for
'pheno.2262' on 'Ovarian cancer || id:bbj−a−139'
